# A Smartphone App (BlueIce) for Young People Who Self-Harm: Open Phase 1 Pre-Post Trial

**DOI:** 10.2196/mhealth.8917

**Published:** 2018-01-30

**Authors:** Paul Stallard, Joanna Porter, Rebecca Grist

**Affiliations:** ^1^ Child and Adolescent Mental Health Group Department for Health University of Bath Bath United Kingdom; ^2^ Child and Family Mental Health, Temple House Oxford Health National Health Service Foundation Trust Keynsham United Kingdom

**Keywords:** self-injury, smartphone, mobile apps, BlueIce, adolescents, cognitive behavioral therapy, mHealth

## Abstract

**Background:**

Recent years have seen a significant increase in the availability of smartphone apps for mental health problems. Despite their proliferation, few apps have been specifically developed for young people, and almost none have been subject to any form of evaluation.

**Objective:**

This study aimed to undertake a preliminary evaluation of a smartphone app (BlueIce), coproduced with young people and designed to help young people manage distress and urges to self-harm. We aimed to assess the acceptability, safety, and use of BlueIce and to explore the effects on the primary outcome of self-harm and the secondary outcomes of psychological functioning.

**Methods:**

We undertook an open trial where we recruited young people aged 12 to 17 years attending specialist child and adolescent mental health services (CAMHS) who were currently self-harming or had a history of self-harm. Eligible participants were assessed at baseline and then given BlueIce. They were assessed 2 weeks later (post familiarization) and again at 12 weeks (post use). A behavior-screening questionnaire (Strengths and Difficulties Questionnaire) was completed along with standardized measures of depression (Mood and Feelings Questionnaire or MFQ) and anxiety (Revised Child Anxiety and Depression Scale or RCADS), taking into account self-reports of self-harm, app helpfulness, and safety.

**Results:**

All core CAMHS professional groups referred at least 1 young person. Out of 40 young people recruited, 37 (93%) elected to use BlueIce after familiarization, with 29 out of 33 (88%) wanting to keep it at the end of the study. No young person called the emergency numbers during the 12-week trial, and no one was withdrawn by his or her clinician due to increased risk of suicide. Almost three-quarters (73%) of those who had recently self-harmed reported reductions in self-harm after using BlueIce for 12 weeks. There was a statistically significant mean difference of 4.91 (*t*_31_=2.11; *P*=.04; 95% CI 0.17-9.64) on postuse symptoms of depression (MFQ) and 13.53 on symptoms of anxiety (RCADS) (*t*_30_=3.76; *P*=.001; 95% CI 6.17-20.90), which was evident across all anxiety subscales. Ratings of app acceptability and usefulness were high.

**Conclusions:**

Our study has a number of methodological limitations, particularly the absence of a comparison group and a prospective way of assessing self-harm. Nonetheless, our findings are encouraging and suggest that BlueIce, used alongside a traditional CAMHS face-to-face intervention, can help young people manage their emotional distress and urges to self-harm.

## Introduction

### Self-Harm

Self-harm has been defined as the intentional self-poisoning or self-injury, irrespective of the type of motive or the extent of suicidal intent [1]. Self-harm in young people aged up to 18 years is a major public health problem [2] with community surveys reporting that up to 20% of young people will self-harm by the age of 18 years [3-7]. Figures from hospital episode statistics show that in 2014-2015, 16,000 young people attended accident and emergency department (A&E) in England following self-poisoning [8], whereas a survey of National Health Service (NHS) trusts found that 19,000 adolescents were hospitalized in England and Wales following self-harm in 2016 [9].This statistic underestimates the scale of the problem because the majority of self-harm remains hidden with comparatively few episodes being treated in a hospital [10].

Although self-harm in young people is common, few intervention studies have been reported [11]. Evidence that is available suggests that therapeutic assessment, mentalization, dialectical behavioral therapy (DBT), and cognitive-behavioral therapy (CBT) may be promising and warrant further evaluation [11]. Key challenges for future research are the development and assessment of innovative interventions that are acceptable to young people, which reduce the risk of self-harm and enhance meaningful engagement with health services [12].

### Digital Health

In the United Kingdom, the NHS is being encouraged to harness digital technology to enhance and support psychological health [13]. Digital health technology may be particularly attractive for young people who are very familiar with and frequent users of digital media. It offers an accessible way of supporting health services because nearly all young people aged 12-15 years have Internet access and 90% own a smartphone [14].

One form of digital technology, smartphone apps, has seen particular growth [15]. However, few apps have been specifically developed for young people with mental health problems, almost none have been subject to any form of evaluation, and none have been developed specifically for children who self-harm [16]. It is essential that smartphone apps for adolescents are subject to research evaluation [16] and codesigned with people who have lived experience [11].

We developed and coproduced a smartphone app (BlueIce) with young people with lived experience of self-harm. BlueIce is to be used in conjunction with face-to-face interventions and provides a personalized toolbox of strategies founded on evidence-based approaches that can be accessed at any time. The aim of this study was to explore the safety, acceptability, feasibility, and usability of a novel smartphone app, BlueIce, developed specifically for young people who self-harm. We will explore the effect on self-harm (primary outcome) and also on psychological functioning (secondary outcome).

## Methods

### BlueIce

We coproduced a smartphone app, BlueIce, with young people with a lived experience of self-harm. We adopted a user-centered, agile development process to create, refine, and evaluate BlueIce. This was a tripartite process whereby the product users (young people and clinical staff), academics, and app developers worked together. This process aimed to maximize the acceptability and use of the app by our target group and ensured that the content reflected current evidence and best clinical practice. Informed by the available evidence, BlueIce provides a personalized toolbox of strategies based on CBT and DBT that can be accessed 24/7 [1,11]. It includes a mood diary, menu of personalized mood-lifting activities, and automatic routing through safety checks to delay or prevent self-harm. Mood-lifting activities are designed to improve mood and include a personalized music library of uplifting music, photo library of positive memories, physical activities, mood-changing activities, audio-taped relaxation and mindfulness exercises, identification and challenging of negative thoughts, a contact list of key people to call or text, and distress tolerance activities (informed by DBT). After using the mood-lifting section, the young person is asked to rerate his or her mood, and if the urge to self-harm has not reduced, that young person is automatically routed to emergency numbers (nominated contact, Childline, 111) that he or she can call.

BlueIce is for young people aged 12-17 years and is designed as an adjunct to face-to-face therapy. It is available for use on Apple and Android devices. It has met the minimum standards required for NHS-accredited apps [17]. BlueIce is password protected and all data are stored locally on the device. BlueIce is a prescribed app and is available via license to child and adolescent mental health services (CAMHS). Information about the app is available at the webpage )[18].

### Study Design

This is a phase 1 open uncontrolled trial. A detailed summary of the methodology is provided in the study protocol [19].

### Setting

Young people were recruited from specialist CAMHS outpatient clinics provided by Oxford Health NHS Foundation Trust. The trust provides CAMHS across Bath and North East Somerset, Buckinghamshire, Oxfordshire, Swindon, and Wiltshire in the United Kingdom.

### Participants

Eligible young people aged 12-17 years who were currently self-harming or had a history of self-harm were included. Young people were excluded if first, they were seriously contemplating or planning a suicide attempt. Given that we do not know whether BlueIce will have any unintentional adverse effects, it would not be safe to test it with a high-risk group who were actively suicidal. Second, young people were excluded if they were diagnosed with psychosis or had a significant learning disability, which might impede their ability to use the app. Third, those who had been subject to abuse within the last 6 months or were the subject of a safeguarding investigation were excluded. Finally, BlueIce is only available in English, and we therefore excluded those who were not proficient in English.

### Recruitment and Consent

Eligible participants were identified by their CAMHS clinician and provided with a project information sheet. Interested young people, and their parents if they are under 16 years, met with a researcher to get familiarized with the project. Signed consent was obtained from young people above the age of 16 years, and signed parent consent and child assent from those under 16 years. The project was reviewed and approved by the South West—Exeter Research Ethics Committee (16/SW/0018).

Consenting participants were provided with BlueIce but continued to attend face-to-face meetings with their CAMHS clinician. As is usual practice, the CAMHS clinician was responsible for reviewing risk and implementing any risk plans required to keep the young person safe.

### Assessments

Standardized questionnaires were completed at baseline, post familiarization (2 weeks after using BlueIce), and post use (12 weeks).

#### Depression

Symptoms of depression were assessed by the Mood and Feelings Questionnaire (MFQ), a self-report questionnaire for depression [20]. The MFQ consists of 33 items, which the young person rates as either *true* (scores 2), *sometimes true* (scores 1) or *not true* (scores 0). The MFQ has high criterion validity and correlates well with other measures of depression [21]. A total score of 27 and above is associated with major depression, 20 with mild depression, and 16 with no mood disorder [21-23].

#### Anxiety

Symptoms of anxiety were assessed by the Revised Child Anxiety and Depression Scale (RCADS) [24]. RCADS is a 47-item questionnaire with items corresponding to Diagnostic and Statistical Manual 4th Edition (DSM-IV) criteria for anxiety in the areas of social phobia, separation anxiety, obsessive compulsive disorder, panic disorder, and generalized anxiety disorder and also for other major depressive disorders. Each item is rated on a 4-point Likert scale of frequency ranging from *never* (0) to *always* (3), and items are then summed to produce subscale and total anxiety scores. There are age- and gender-related norms for identifying clinically significant scores (total score≥64-80) [24]. If the young person was under 16 years, his or her parents were also asked to complete this measure.

#### Behavior

The Strengths and Difficulties Questionnaire (SDQ) is a brief, widely used behavioral screening questionnaire [25]. The SDQ consists of 25 items that assess emotional symptoms, conduct problems, hyperactivity and/or inattention, peer relationship problems, and prosocial behavior. Participants also rate overall distress and social impact of their behavior on home life, friendships, classroom learning, and leisure activities. Parents also completed this measure if the young person was under 16 years.

#### Safety

At post familiarization, young people were asked whether BlueIce makes them feel like harming themselves even more, whether they felt able to use BlueIce if they had thoughts of self-harm, and whether they thought BlueIce would help them stop self-harming. Participants rated their responses on a 5-point Likert scale ranging from 1 (*definite no*) to 5 (*definitely*).

#### Acceptability

At post use, young people used a 10-point Likert scale to rate the ease of use, helpfulness, and whether they would recommend BlueIce to a friend.

#### Self-Harm

Information on baseline levels of self-harm was obtained from clinical records spanning the 4 weeks before using BlueIce. Self-harm during the trial was assessed via self-report during the interview at baseline and 12 weeks. By estimating the number of self-harm acts that would have occurred if participants continued to self-harm at the same rate through the 12-week trial, we calculated the number of self-harm acts prevented for the young people who (1) stopped self-harming at post use and (2) continued to self-harm at post use but at a reduced rate.

## Results

### Demographics and Baseline Assessments

Between May and October 2016, 37 different clinicians from 8 CAMHS teams referred 54 young people. All core professionals that constitute CAMHS, that is, child psychiatrists, clinical psychologists, family therapists, child psychotherapists, occupational therapists, and community psychiatric nurses, referred at least 1 young person. Of the 54 referrals, 4 did not meet inclusion criteria, 3 we were unable to contact, 2 dropped out of CAMHS before they could consent, and 1 declined to participate. The remaining 44 completed baseline assessments.

Participants were predominantly girls (40, 91%) with an average age of 16.0 years (SD=1.4), with 30 (68%) having self-harmed at least once in the 4 weeks before starting the trial.

Using recommended cut-offs, 42 young people out of 44 (96%) scored 29 or more on the MFQ, suggesting probable depression. Using age- and gender-adjusted cut-offs on the RCADS, 37 out of 44 (84%) screened positive for one or more anxiety disorders and 37 out of 44 (84%) scored above cut-offs on the SDQ for a probable emotional disorder. On the RCADS, 16 out of 17 (94%) parents rated their child above the cut-off for depression and 16 out of 18 parents (89%) scored their child above the cut-off on the SDQ for significant emotional problems. Demographic data and baseline psychopathology are summarized in Tables 1 and 2.

Data summarized in Table 2 indicate that both young people and parents identified significant problems (young people=85%; parents=95%), which had been present for longer than 12 months (young people=85%; parents=95%) and caused significant distress (young people=78%; parents=100%). The majority of young people reported significant impairment in friendships (80%), ability to learn (76%), leisure activities (73%), and home life (61%).

### Post Familiarization

Postfamiliarization assessments were completed with 40 of the 44 (91%) participants, and of these, 37 (93%) elected to use BlueIce. Those who did not want to use BlueIce reported that they were not ready to stop self-harming. Table 3 summarizes postfamiliarization data.

No safety issues were identified and there were no unintended negative effects on self-harm. No young person thought that BlueIce would increase his or her self-harm with 32 out of 40 (80%) strongly endorsing this statement.

In total, 33 out of 40 (83%) thought they would be able to use BlueIce if they had thoughts of self-harm. Only 2 (5%) felt they would be unable to do so feeling that their urges to self-harm would be too powerful to resist. Young people were less sure about whether BlueIce would help them to stop self-harming, 24 (60%) thought it would, 8 (20%) were unsure, and 6 (15%) felt it might, with 2 (5%) feeling it would not.

### Post Use

Postuse assessments were completed with 33 out of the 37 (89%) young people, with paired data being available for 30-32 young people according the specific items analyzed. Data are summarized in Table 4.

**Table 1:** Characteristics of participants.

Characteristic		Number of participants, n
**Gender**		
	Male	4
	Female	40
**Age (years)**		
	12	1
	13	5
	14	3
	15	9
	16	15
	17	11
**Self-harm**		
	Self-harmed at least once in past 4 weeks	30
	No self-harm in last 4 weeks	14

**Table 2:** Young person and parent-rated baseline symptomatology.

Measure	Child report	Parent report
Mood and Feelings Questionnaire, mean (SD)	43.6 (9.6)	N/A^a^
Revised Child Anxiety and Depression Scale, mean (SD)	81.0 (21.9)	75.4 (26.9)
Strengths and Difficulties Questionnaire, mean (SD)	21.4 (3.3)	23.8 (6.3)
Definite or severe problem, n (%)	33 (85)	17 (95)
Problem present longer than 12 months, n (%)	33 (85)	17 (95)
Causes medium to great deal of distress, n (%)	31 (78)	18 (100)
Effect on home life (medium to great deal), n (%)	23 (61)	14 (78)
Effect on friendships (medium to great deal), n (%)	31 (80)	16 (89)
Effect on ability to learn (medium to great deal), n (%)	28 (76)	11 (61)
Effect on leisure (medium to great deal), n (%)	29 (73)	14 (78)
Burden on you and family (quite a lot to great deal), n (%)	31 (80)	15 (83)

^a^N/A: not applicable.

**Table 3:** BlueIce post familiarization (2 weeks).

Question	No	Maybe	Not sure	Think so	Definitely
Would you be able to use BlueIce if you had thoughts of self-harm?	2	2	3	20	13
Do you think BlueIce might make you harm more?	32	4	4	0	0
Do you think BlueIce would help you to stop harming?	2	6	8	15	9

**Table 4:** Paired baseline and follow-up scores on standardized measures.

Measure			Baseline, mean (SD)	Follow-up, mean (SD)	Significance	
					*t* (degrees of freedom)	*P* value
**Self-report (n=30-32)**						
	Mood and Feelings Questionnaire (MFQ)		42.75 (10.73)	37.84 (15.44)	2.11 (31)	.04
	**Revised Child Anxiety and Depression Scale (RCADS)**					
		Panic disorder	14.00 (7.31)	11.20 (6.40)	2.90 (29)	.007
		Separation anxiety disorder	8.90 (4.20)	7.37 (4.97)	2.77 (28)	.01
		Generalized anxiety disorder	11.27 (3.50)	9.50 (4.05)	2.72 (29)	.01
		Social anxiety disorder	19.67 (5.65)	16.60 (6.33)	3.58 (29)	.001
		Obsessive compulsive disorder	6.97 (4.21)	5.70 (4.74)	2.64(29)	.01
		Depression	19.16 (5.13)	16.58 (6.62)	2.40 (30)	.02
		Total RCADS	80.33 (23.75)	66.80 (28.46)	3.76 (29)	.001
	**Strengths and Difficulties Questionnaire (SDQ)**					
		Hyperactivity scale	5.44 (1.63)	5.22 (1.83)	0.62 (31)	.54
		Emotional symptoms scale	7.91 (1.51)	7.06 (2.17)	2.90 (31)	.007
		Peer problems scale	4.91 (1.55)	5.25 (1.61)	−1.36 (31)	.18
		Prosocial scale	7.34 (2.24)	7.63 (1.56)	−1.01 (31)	.32
		Conduct problems scale	2.91 (1.23)	2.75 (1.05)	0.67 (31)	.51
		Total SDQ	21.16 (3.35)	20.28 (4.47)	1.16 (31)	.26
**Parent-report (n=10-13)**						
	**Revised Child Anxiety and Depression Scale (RCADS)**					
		Panic disorder	13.70 (6.36)	12.10 (4.04)	1.50 (9)	.17
		Separation anxiety disorder	10.73 (5.29)	9.91 (4.74)	1.22 (10)	.25
		Generalized anxiety disorder	11.55 (4.74)	10.00 (2.93)	1.76 (10)	.11
		Social anxiety disorder	18.64 (5.56)	17.64 (6.14)	1.08 (10)	.31
		Obsessive Compulsive Disorder	9.10 (5.86)	9.50 (5.15)	0.58 (9)	.59
		Depression	17.91 (6.49)	17.64 (6.35)	0.27 (10)	.79
		Total RCADS	80.90 (30.49)	77.70 (23.21)	1.03 (9)	.33
	**Strengths and Difficulties Questionnaire (SDQ)**					
		Hyperactivity scale	4.31 (1.65)	4.15 (1.91)	0.41 (12)	.69
		Emotional symptoms scale	8.17 (2.37)	7.25 (2.60)	2.42 (11)	.03
		Peer problems scale	8.38 (3.82)	5.77 (1.79)	2.50 (12)	.03
		Prosocial scale	7.23 (1.64)	6.54 (1.85)	1.03 (12)	.32
		Conduct problems scale	2.92 (1.12)	3.23 (1.83)	0.72 (12)	.49
		Total SDQ	24.58 (6.95)	20.50 (6.25)	4.67 (11)	.001

**Table 5:** Self-harm status at baseline and post use.

Self-harm status		Number of participants, n
**At baseline**		
	Not self-harmed in 4 weeks before baseline assessment	7
	Self-harmed in 4 weeks before baseline assessment	26
**Post use (12-week)**		
	Not self-harmed at follow-up	7
	Self-harmed during follow-up	0
	Not self-harmed during follow-up	4
	Self-harmed during follow-up at a reduced rate	15
	Self-harmed during follow-up at same rate	7

#### Depression, Anxiety, and Behavior

At follow-up, there was a statistically significant mean difference of 4.91 (*t*_31_=2.11; *P*=.04; 95% CI 0.17-9.64) on postuse symptoms of depression (MFQ) and 13.53 on symptoms of anxiety (RCADS) (*t*_30_=3.76; *P*=.001; 95% CI 6.17-20.90), which was evident across all anxiety subscales. There was no statistically significant change on the behavior-screening questionnaire (SDQ) other than a significant mean difference of 0.84 on the emotional subscale (*t*_29_=2 .90; *P*=.007; 95% CI 0.25-1.44).

The analysis was repeated comparing those who reported no change in self-harm (n=7) with those who had not harmed or did so at a reduced rate (n=26). There were no postuse changes on any measure for those who self-harmed at the same rate. For those who had not harmed or did so at a reduced rate, there was a 7.48 postuse reduction on mean MFQ (95% CI 1.94-13.03; *t*_24_=2.78; *P*=.01), 16 mean reduction on the RCADS (95% CI 7.63-24.37; *t*_24_=3.95, *P*=.001), and 1.0 mean reduction on the emotional subscale of the SDQ (95% CI 0.27-1.73, *t*_24_=2.81, *P*=.01).

A total of 18 parents of younger children completed baseline measures. Of these, 13 completed postuse questionnaires with paired data being available for 10-13 parents according to the specific items analyzed. There were no significant differences on the parent-completed RCADS. However, on the SDQ, there was a statistically significant postuse mean difference of 0.92 on the emotional subscale (95% CI 0.083-1.75; *t*_11_=2.42; *P*=.03), 2.61 on the peer relationship subscale (95% CI 0.334-4.90; *t*_12_=2.50; *P*=.03), and 4.08 on the total score (95% CI 2.16-6.01; *t*_11_=4.67; *P*=.001).

Numbers were too small to compare parental reported changes in symptoms for young people who reported changes in self-harm with those who were self-harming at the same rate.

#### Acceptability

Finally, postuse ratings on a 10-point scale (higher score=more positive endorsement) were high for ease of use (8.9, SD=1.2) and whether they would recommend BlueIce (8.6, SD=1.6) and slightly lower for helpfulness (6.6, SD=2.2). At the end of the study, 29 of the 33 interviewed (88%) wanted to keep the app. Of those who did not want it, 1 no longer felt she needed it, 2 were not ready to stop self-harming, and 1 felt it was too much of a chore to use.

### Self-Harm

Self-report changes in self-harm are summarized in Table 5.

All of those who reported not self-harming in the 4 weeks before baseline assessment maintained their status and had not self-harmed over the course of the 12-week trial. Of those 26 who had self-harmed at baseline, 4 (15%) had completely stopped, with a further 15 (58%) reporting less frequent acts of self-harm at follow-up. There was a small group of young people (7, 27%) who reported no reductions in their self-harming behavior over the 12-week trial.

We calculated the number of self-harm acts prevented in 2 ways. First, we obtained data on baseline rates of self-harm for the 4 young people who had stopped self-harming at follow-up. We used this to estimate the number of self-harm events that were prevented if they continued to self-harm at the same rate, during the 12-week study. Second, for the 15 who continued to self-harm but at a reduced rate, we quantified the change to determine how many episodes BlueIce had prevented. Of the 19 young people who reported reduced self-harm, 10 had a reduction of 1-5 incidents, 2 a reduction of 6-10 incidents, and 7 a reduction of 11 or more incidents. These calculations suggest that a total of 308 incidents of self-harm were prevented during the course of this study.

Finally, feedback from young people at the end of the study revealed that no one had called the emergency numbers provided on BlueIce. Similarly, no clinician withdrew any young person from the study due to an escalation of risk or the emergence of suicidal planning or a suicide attempt.

## Discussion

### Principal Findings

Our study group comprised young people with chronic mental health problems that were significantly impairing most parts of their everyday life. They reported high levels of symptoms of depression and anxiety, with over two-thirds self-harming at least once in the 4 weeks before joining the study. It is therefore encouraging that a majority of this group found BlueIce to be acceptable and were keen to use it after familiarization and elected to keep it at the end of the study. Similarly, BlueIce proved acceptable to all core professionals groups working in CAMHS. Child and adolescent psychiatrists, clinical psychologist, family therapists, child psychotherapists, nurses, and occupational therapists all referred young people to the project. Statistically significant reductions in symptoms of depression and anxiety over the course of the 12-week trial are also encouraging.

### Codesign

The acceptability of BlueIce was undoubtedly enhanced by the involvement of young people and clinicians during the design and development phase. The app was coproduced with young people who advised on all aspects including design, layout, flow, and content. Similarly, workshops with clinicians ensured that the mood-lifting techniques reflected both the recommended interventions of the National Institute for Health and Care Excellence and clinical practice. Our experience supports the recommendation of a recent review that any new therapeutic intervention should be developed in collaboration with patients to ensure that it meets their needs [11].

### Adverse Effects

mHealth researchers have reported that apps can pose risks to patient safety and that actions should be taken to mitigate these risks [26,27]. Given the nature of self-harm and associated comorbid difficulties, it was necessary to ensure BlueIce worked as intended and did not make young people’s difficulties worse. There were no adverse events during the course of the study. No young person used the emergency contact numbers, none were withdrawn by their clinician following increased risk, and young people did not feel that BlueIce would increase their self-harm. Young people were more cautious when answering about whether BlueIce would help them to stop self-harming. Although only a few completely stopped, almost three-quarters reported that they had stopped or were self-harming at a reduced rate after using BlueIce. These young people identified a total of 308 potential episodes of self-harm that were prevented over a 12-week period. Although the majority of self-harm does not result in emergency department attendance, it is likely that some of these episodes would have resulted in contact with primary or secondary care services. Furthermore, the use of BlueIce may also reduce the number of face-to-face contacts with mental health services. This needs to be examined in future studies as the potential cost savings from this low-cost intervention could be considerable.

### Who Did Not Find BlueIce Helpful

There were a small minority of young people who did not find BlueIce helpful. The 3 who declined to use BlueIce after familiarization reported that they did not feel ready to stop self-harming. Similarly, feedback from those who used BlueIce suggested that there were times when their distress was too overwhelming to use the app. Our findings suggest that the preparedness of young people to stop self-harming needs careful assessment. Providing BlueIce for young people who are not motivated or ambivalent to change will not be effective. Second, young people need to be encouraged to use BlueIce at an earlier stage in the distress escalation cycle. By earlier use, they may be able to prevent the distress buildup and self-harm cycle. However, we are mindful that BlueIce is a smartphone app and that the limitations of digital support in preventing all self-harm need to be recognized.

### Limitations

Our study is exploratory and as such has a number of limitations. First, we report a self-selected case series of young people who actively elected to trial BlueIce. Our group may, therefore, have been more motivated and prepared to address their self-harm. Second, this was an open uncontrolled study, and we did not have a comparison group. We do not know whether the improvements we report are better or worse than usual care. Third, BlueIce was provided in addition to usual face-to-face intervention. Consequently, we do not know whether the improvements on symptoms of depression, anxiety, and reduction in self-harm we noted were due to BlueIce or their face-to-face intervention. A randomized trial comparing treatment as usual with and without BlueIce is required to quantify the benefits of adding BlueIce to usual care.

Finally, we assessed self-harm through retrospective self-reports, which may be subject to recall bias. Although this limitation is recognized, young people were clearly able to identify changes in their self-harm during the interview, and they directly attributed these to the use of BlueIce rather than to the usual care they had received. Subsequent studies should explore the use of prospective self-harm diaries and the exploration of hospital records of A&E attendances following self-harm.

Finally, we are reporting a small exploratory study that is not sufficiently powered to explore the effects on psychological functioning or self-harm over time. Although our results are promising, we need to be cautious about the strength of any conclusions that can be drawn from our findings. This needs to be addressed in a subsequent, suitably powered, randomized controlled trial.

### Conclusions

In spite of these limitations, our results are encouraging and suggest that a smartphone app, used alongside a traditional CAMHS face-to-face intervention, can help young people manage their emotional distress and urges to self-harm. BlueIce proved highly acceptable to young people and clinicians. It was safe with young people reporting a number of improvements in their mood and reductions in self-harm after use. Further research is required to determine the additional benefits of adding BlueIce to face-to-face care and in quantifying potential cost savings that may result from fewer emergency department attendances. However, in the meantime, as an adjunct to usual face-to-face care, BlueIce appears an acceptable and readily accessible way of empowering young people to manage their distress.

## Abbreviations

CAMHS: child and adolescent mental health services
CBT: cognitive-behavioral therapy
DBT: dialectical behavioral therapy
MFQ: Mood and Feelings Questionnaire
NHS: National Health Service
RCADS: Revised Child Anxiety and Depression Scale
SDQ: Strengths and Difficulties Questionnaire

## References

[1] National Collaborating Centre for Mental Health (2011). NICE.

[2] Hawton K, Saunders KEA, O'Connor RC (2012). Self-harm and suicide in adolescents. Lancet.

[3] Kidger J, Heron J, Lewis G, Evans J, Gunnell D (2012). Adolescent self-harm and suicidal thoughts in the ALSPAC cohort: a self-report survey in England. BMC Psychiatry.

[4] Hawton K, Rodham K, Evans E, Weatherall R (2002). Deliberate self harm in adolescents: self report survey in schools in England. Br Med J.

[5] Madge N, Hewitt A, Hawton K, de Wilde EJ, Corcoran P, Fekete S, van Heeringen K, De Leo D, Ystgaard M (2008). Deliberate self-harm within an international community sample of young people: comparative findings from the Child & Adolescent Self-harm in Europe (CASE) Study. J Child Psychol Psychiatry.

[6] Klonsky ED, Muehlenkamp JJ (2007). Self-injury: a research review for the practitioner. J Clin Psychol.

[7] Stallard P, Spears M, Montgomery AA, Phillips R, Sayal K (2013). Self-harm in young adolescents (12-16 years): onset and short-term continuation in a community sample. BMC Psychiatry.

[8] Campbell D (2016). Theguardian.

[9] (2016). NSPCC.

[10] McMahon EM, Keeley H, Cannon M, Arensman E, Perry IJ, Clarke M, Chambers D, Corcoran P (2014). The iceberg of suicide and self-harm in Irish adolescents: a population-based study. Soc Psychiatry Psychiatr Epidemiol.

[11] Hawton K, Witt KG, Taylor Salisbury TL, Arensman E, Gunnell D, Townsend E, van Heeringen K, Hazell P (2015). Interventions for self-harm in children and adolescents. Cochrane Database Syst Rev.

[12] Hawton K, Saunders KE, O'Connor RC (2012). Self-harm and suicide in adolescents. Lancet.

[13] NHS (2015). Gov.uk.

[14] (2014). Ofcom.

[15] Anthes E (2016). Mental health: there's an app for that. Nature.

[16] Grist R, Porter J, Stallard P (2017). Mental health mobile apps for preadolescents and adolescents: a systematic review. J Med Internet Res.

[17] Developer Developer.nhs.uk.

[18] Oxford Health NHS Foundation Trust Oxfordhealth.nhs.uk.

[19] Stallard P, Porter J, Grist R (2016). Safety, acceptability, and use of a smartphone app, BlueIce, for young people who self-harm: protocol for an open phase I trial. JMIR Res Protoc.

[20] Costello EJ, Angold A (1988). Scales to assess child and adolescent depression: checklists, screens, and nets. J Am Acad Child Adolesc Psychiatry.

[21] Daviss WB, Birmaher B, Melhem NA, Axelson DA, Michaels SM, Brent DA (2006). Criterion validity of the Mood and Feelings Questionnaire for depressive episodes in clinic and non-clinic subjects. J Child Psychol Psychiatry.

[22] Kent L, Vostanis P, Feehan C (1997). Detection of major and minor depression in children and adolescents: evaluation of the Mood and Feelings Questionnaire. J Child Psychol Psychiatry.

[23] Wood A, Kroll L, Moore A, Harrington R (1995). Properties of the Mood and Feelings Questionnaire in adolescent psychiatric outpatients: a research note. J Child Psychol Psychiatry.

[24] Chorpita BF, Moffitt CE, Gray J (2005). Psychometric properties of the Revised Child Anxiety and Depression Scale in a clinical sample. Behav Res Ther.

[25] Goodman R (1997). The Strengths and Difficulties Questionnaire: a research note. J Child Psychol Psychiatry.

[26] Lewis TL, Wyatt JC (2014). mHealth and mobile medical Apps: a framework to assess risk and promote safer use. J Med Internet Res.

[27] Naeem F, Gire N, Xiang S, Yang M, Syed Y, Shokraneh F, Adams C, Farooq S (2016). Reporting and understanding the safety and adverse effect profile of mobile apps for psychosocial interventions: an update. World J Psychiatry.

